# Spatiotemporal gait parameters and ground reaction force analysis in female non-gymnasts *versus* gymnasts during adolescence and university stages

**DOI:** 10.3389/fbioe.2026.1839156

**Published:** 2026-09-22

**Authors:** Amany E. Abd-Eltawab, Yusuf Saleem Khan, Eslam K. Fahmy, Mustafa Shukry, Dalia Mahmoud Abdelmonem Elsherbini, Hailah M. Almohaimeed, Safya E. Esmaeel, Aisha Farhana

**Affiliations:** 1 Department of Physical Therapy and Health Rehabilitation, College of Applied Medical Sciences, Jouf University, Sakaka, Saudi Arabia; 2 Department of Anatomy, College of Medicine, University of Hail, Hail, Saudi Arabia; 3 Department of Physiology, College of Medicine, Northern Border University, Arar, Saudi Arabia; 4 Department of Biomedical Sciences, College of Veterinary Medicine, King Faisal University, Al-Ahsa, Saudi Arabia; 5 Department of Clinical Laboratory Sciences, College of Applied Medical Sciences, Jouf University, Sakaka, Saudi Arabia; 6 Department of Basic Sciences, College of Medicine, Princess Nourah bint Abdulrahman University, Riyadh, Saudi Arabia

**Keywords:** ground reaction force, gymnast, rehabilitation, spatiotemporal gait parameters, sports

## Abstract

**Background and Aim:**

Gymnastics requires balance, coordination, and flexibility, making it both enjoyable and challenging. However, ensuring safety is crucial to prevent injuries. This study aimed to assess key spatiotemporal gait parameters, including cadence, velocity, stride length, cycle time, and step width, in conjunction with the anteroposterior ground reaction force (GRF-X) across five sub-phases of the gait cycle, in female gymnasts and non-gymnasts during the adolescence and university stages.

**Methods:**

In this study, 40 participants were recruited and categorized into two primary groups based on age: Group I represented the adolescent stage and comprised female middle school students, and Group II represented the university stage and comprised female university students. Each primary group was further divided into two subgroups: non-gymnasts and gymnasts. Therefore, four distinct groups were formed: 10 non-gymnast female middle school students, 10 gymnast female middle school student, 10 non-gymnast female university students, and 10 gymnast female university students. Participants were matched for age, height, and weight. A two-way multivariate ANOVA (MANOVA) was performed to evaluate the effects of group (junior *vs.* university) and activity (gymnast *vs.* non-gymnast) on the combined dependent variables, including cadence, walking speed, stride length, cycle time, step width, and GRF-X, across the gait sub-phases. A *p*-value of ≤ 0.05 was considered statistically significant.

**Results:**

The results indicated significant differences between female middle school gymnasts and non-gymnasts in cadence, speed, cycle time, and step width. Similarly, significant differences were identified between female university students who were gymnasts and those who were not, specifically in cadence, speed, and stride length. Furthermore, the mean values of GRF-X during the initial contact, loading response, and mid-stance phases differed significantly between female middle school gymnasts and non-gymnasts. Additionally, a significant difference was noted between gymnast and non-gymnast female university students during the initial contact and mid-stance phases.

**Conclusion:**

The results revealed significant alterations in the GRF-X values across the four groups. Furthermore, the cadence, speed, cycle time, and step width exhibited significant differences between the non-gymnast and gymnast groups; therefore, it is recommended to strengthen lower limb muscles, normalize gait parameters, and improve ankle range of motion to mitigate injury risk. Normal gait provides a fundamental benchmark for subsequent comparisons with task-specific activities.

## 1 Introduction

The analysis of spatiotemporal gait parameters in adolescent female gymnasts is a well-established method in sports science for assessing biomechanical performance and injury risk. This study compared gymnasts and non-gymnasts to highlight the biomechanical differences that may affect susceptibility to injuries. Gymnasts frequently execute highly complex and precise movements, which inherently elevate the risk of sports-related injuries (Semenov et al., 2021).

As a sport, gymnastics requires both maintenance of body control and adequate lower limb explosive force. Advancements in gymnastics necessitate the continual development of new and more difficult skills, as well as improved performance ratings in competitions (Sleeper et al., 2012; Hall et al., 2016). Flexibility, speed, power, agility, muscular strength, balance, coordination, proprioception, and postural control are all important training attributes that determine the success of gymnasts (Bobo-Arce and Méndez-Rial, 2013; Gateva, 2019). These physical qualities enable gymnasts to execute complex acrobatic skills involving running, jumping, landing, and rapid changes in body position while maintaining efficiency and dynamic stability (Sands et al., 2011; Sleeper et al., 2012). The continuous execution of these demanding skills exposes gymnasts to substantial mechanical loading and requires efficient force generation, shock absorption, and neuromuscular control to prevent injury.

Consequently, the assessment of spatiotemporal gait parameters and ground reaction forces (GRFs) provides valuable insights into locomotor performance and lower limb loading patterns. External gait parameters are crucial for determining movement efficiency and overall performance (Potop et al., 2014; Dallas and Theodorou, 2018; Błażkiewicz et al., 2019; Cerasela et al., 2020). Spatiotemporal gait parameters, including cadence, gait speed, stride length, gait cycle duration, and step width, reflect movement efficiency, dynamic balance, and lower limb motor control, whereas GRFs quantify the braking and propulsive forces generated during gait. During gymnastics landings, peak GRFs have been reported to reach approximately 7.1–15.8 times the body weight, placing considerable stress on the ankle joint and surrounding soft tissues and increasing the risk of musculoskeletal injuries (Wade et al., 2012; Slater et al., 2015). Therefore, evaluating spatiotemporal gait characteristics together with GRFs provides an objective assessment of locomotor performance, lower limb loading patterns, and injury risk in gymnasts.

Similarly, Gómez-Landero et al. (2021) and Asseman et al. (2008) found that gymnast expertise enhances postural stability, as evidenced by improved control of the center of pressure (COP). Specific tasks in sports are highly and reflect the level of expertise of the gymnasts (Marcolin et al., 2019). Gymnasts have greater postural stability than non-gymnasts (Carrick et al., 2007), with reduced COP displacement and superior proprioceptive reintegration. Furthermore, advanced gymnast skills correlate with more stable and less variable acceleration time series (Lamoth et al., 2009). Gymnastics training also improves the ability to adapt frames of reference, influencing gait parameters during normal walking (Croix et al., 2010).

Čeklić and Šarabon (2021) investigated differences in isometric muscle strength of the lower limbs, specifically the hip, knee, and ankle, between gymnasts and non-gymnasts (Kyselovičová and Zemková, 2010; Čeklić and Šarabon, 2021). Fifteen gymnasts and 15 non-gymnasts performed unilateral isometric voluntary contractions of the hip, knee, and ankle agonists and antagonists using a dynamometer. Inter-limb asymmetries were calculated based on strength measurements. The results indicated significant differences between the two groups, with gymnasts exhibiting superior strength only in the hip strength parameters. These findings highlight the distinct strength profiles of gymnasts, particularly in the hip muscle.

The stability of the standing posture is strongly correlated with the athlete’s level of expertise, as observed in running. Senior athletes exhibit markedly superior control of body position compared with junior athletes (Omorczyk et al., 2021). Kinetic and kinematic analyses of gymnasts are key areas of research, especially during different running modalities, including simple and chassé runs (Cicchella, 2009). A study examined the performance of a 12-year-old gymnast who had been practicing gymnastics for 4 years, focusing on the run-up, take-off, and swing phases of a jump leap. The analysis indicated that the angle of flexion–extension during the jump was greater in the simple run. Additionally, the time of foot contact and the period immediately following pre-swing showed higher angular acceleration during leg flexion (Coppola et al., 2020). These findings offer valuable insights into the biomechanical differences between running modalities and their effects on gymnast performance.

Budiarti et al. (2022) studied differences in power and flexibility between male and female junior gymnasts, highlighting gender-based disparities. In this study, 26 boys and 26 girls participating in aerobic gymnastics were assessed using the side split test for flexibility and vertical jump test for explosive power. The results revealed that girls exhibited greater flexibility, whereas boys generated more power. This variation in power, a key kinetic variable, likely contributed to the differences in GRF observed by Budiarti et al. (2022), who attributed part of this variation to gender differences.

There is a lack of comprehensive literature exploring the differences in functional ambulation parameters between young female gymnasts and non-gymnast female university students. There is a lack of data regarding young female cohorts, specifically those aged 11–14 and 20–24 years, which affects the understanding of the effects of aging on gait. The only two studies that included a broad age spectrum and used the GAITRite walkway system were conducted on healthy Asian and Swiss populations; however, neither compared non-gymnasts with gymnasts (Rössler et al., 2024). To the best of our knowledge, no data from the Middle East region have addressed this topic. Comprehensive data are necessary to develop an understanding of the timing and mechanisms of gait changes. These parameters are crucial as they indicate the mechanical load exerted on lower limb joints during walking in both athletic and non-athletic populations (Gogoi et al., 2021). Thus, the results will help highlight the importance of the early identification of load concentration patterns during different activities, especially for gymnasts, who are exposed to repetitive stress.

Therefore, this study aimed to address this gap by evaluating and comparing spatiotemporal gait parameters—including cadence, gait speed, stride length, gait cycle duration, and step width—together with GRF-X characteristics, between gymnasts and non-gymnasts across two female age cohorts (11–14 and 20–24 years). We hypothesized that trained female gymnasts would exhibit superior gait performance, characterized by higher cadence, greater gait speed, longer stride length, shorter gait cycle duration, a narrower base of support, and distinct anteroposterior braking and propulsive GRF profiles, compared with non-gymnasts in both age cohorts.

## 2 Materials and methods

### 2.1 Study design

A comparative analytical cross-sectional research design was used. The study involved determining and comparing functional ambulation and kinetic parameters, specifically cadence, velocity, stride length, base of support, and GRF value in the X-direction, between the study groups.

### 2.2 Participants

A total of 40 participants were recruited for this study and categorized into two age-based groups: Group I represented the adolescent stage and comprised female middle school students, and Group II represented the university stage and comprised female university students. Each group was further divided into two subgroups: non-gymnasts and gymnasts. Consequently, four distinct groups were formed: 10 female middle school students non-gymnast female individuals, 10 female middle school students gymnast female individuals, 10 non-gymnast female university students, and 10 gymnast female university students. The middle school (adolescent) group included participants aged 11–14 years, and the university group included those aged 20–24 years. The participants’ weights ranged from 45 to 60 kg, and their heights ranged from 143 to 160 cm. All gymnasts, both adolescent (middle school) and university level, were affiliated with the Ahly Club Gymnast Center in Cairo, Egypt. The training program was conducted 5 days per week, with each session lasting between 30 and 45 min. The training program consisted of ballet, dynamic warm-up, equipment and body technique, routine practice to refine technical execution, conditioning exercises, and flexibility training (Batista et al., 2018). According to Ma et al. (2024), this training program provides extra advantages for enhancing vertical jumping abilities in addition to jumping interval training. Training was conducted at moderate-to-vigorous intensity under the supervision of certified coaches. The participants’ training experience ranged from 2 to 4 years, with an average of 3.00 ± 0.83 years. Information regarding their training was obtained through pre–data collection interviews. The inclusion criteria for non-gymnast middle school and university students required them to be female individuals who did not engage in sports activities beyond routine daily tasks. The exclusion criteria for all participants included a history of musculoskeletal disorders, previous surgeries involving lower limb joints, or prior trauma to these joints. This rigorous selection process was implemented to ensure a homogeneous sample, facilitating the comparison of functional differences in gait parameters between gymnasts and non-gymnasts.

The sample size was estimated using G*Power software (version 3.1.9.7). An *a priori* power analysis was performed for an F-test (ANOVA: fixed effects, special, main effects, and interactions) corresponding to the 2 × 2 factorial study design. The effect size (Cohen’s f = 0.88) was derived from the cadence results obtained in a pilot study involving 20 participants (five per group) using the same protocol, outcome measures, and participant characteristics as those of the main study. Assuming a significance level of α = 0.05 and statistical power of 90%, the minimum required sample size was estimated to be 35 participants. A 10% dropout rate was assumed to allow for possible attrition in the study. Therefore, the final sample consisted of 40 participants (10 per group).

### 2.3 Instruments

The present study primarily involved the GAITRite® electronic walkway system, and kinetic data were collected using a Qualisys three-dimensional (3D) motion capture system (Qualisys AB, Gothenburg, Sweden) synchronized with an AMTI force platform (Advanced Mechanical Technology Inc., Watertown, MA, United States).

#### 2.3.1 The GAITRite system

Spatiotemporal gait parameters were assessed using the GAITRite® electronic walkway system (CIR Systems Inc., Sparta, NJ, United States), a portable pressure-sensitive walkway embedded with an array of pressure-activated sensors that records the timing and position of each foot contact during walking. Before data collection, the system was calibrated following the manufacturer’s instructions. The GAITRite system was used in conjunction with the Microsoft Kinect Sensor (Microsoft Corp., Redmond, WA, United States) and Motognosis Lab software (Motognosis GmbH, Berlin, Germany) to monitor and process the participants’ gait performance during testing. Participants walked barefoot at a self-selected, comfortable speed along a straight walkway incorporating an approximately 2-m GAITRite sensing area. To minimize acceleration and deceleration effects, the participants started walking before reaching the sensing area and continued walking beyond its end. Three successful walking trials were recorded for each participant, and the average values were used for the analysis. The recorded footfall data were processed using GAITRite software to calculate spatiotemporal gait parameters, including cadence (steps/min), gait velocity (cm/s), stride length (cm), gait cycle duration (min), and step width (cm). The validity and reliability of the GAITRite system for measuring spatiotemporal gait characteristics have been established previously (Hume et al., 1993; Webster et al., 2005).

#### 2.3.2 Qualisys motion capture system

Kinetic gait data were collected using a Qualisys 3D motion capture system synchronized with an AMTI force platform. The system consisted of six high-speed infrared Pro-Reflex cameras and a force platform (60 × 40 cm) embedded at the center of the walkway. The cameras and force platform were synchronized for the simultaneous acquisition of lower limb kinematics and GRF data during each walking trial.

Before testing, the motion capture system was calibrated following the manufacturer’s recommendations to obtain an accurate 3D reconstruction of the marker trajectories within the capture volume (Senior, 2004). Anatomical landmarks were marked with 20 passive reflective markers based on the Qualisys lower-body marker protocol (Qualisys AB, 2011; Elhafez et al., 2019) using double-sided adhesive tape. The bilateral acromion processes, 12th thoracic vertebra, sacrum, bilateral anterior–superior iliac spines, greater trochanters, suprapatellar regions, knee joint lines, tibial tuberosities, ankle joints, heels, and heads of the second and third metatarsals were marked.

Kinetic data were collected using a force platform at 120 Hz. GRF signals were filtered using a fourth-order low-pass Butterworth filter with a cutoff frequency of 10 Hz, and marker trajectory data were filtered using a fourth-order low-pass Butterworth filter with a cutoff frequency of 6 Hz (Robertson and Dowling, 2003). The mean anteroposterior GRF (GRF-X; in N/kg) was extracted from the synchronized recordings and compared between the groups (gymnast *vs.* non-gymnast).

### 2.4 Procedure

The participants were directed to walk normally and naturally along the GAITRite walkway without target walking. Each participant completed three barefoot walking trials along the walkway to ensure consistency in walking pattern and to accurately determine differences in spatiotemporal gait parameters among the groups. Additionally, the Qualisys motion capture system was used in the present study in conjunction with a force plate component to collect kinetic data. The force plate, positioned at the center of the walkway, recorded GRF-X. Participants were asked to walk barefoot over the force plate without any specific targets to maintain natural walking conditions. This setup allowed for the precise measurement of kinetic forces and provided valuable insights into gait dynamics. The duration of barefoot walking was standardized for all groups.

### 2.5 Statistical analysis

Data were analyzed using GraphPad Prism (version 9; GraphPad Software, San Diego, CA, United States) and IBM SPSS Statistics. Data are presented as mean ± SEM. The Shapiro–Wilk test was used to assess the normality of the data distribution. Baseline demographic characteristics (age, weight, height, and body mass index) were compared between gymnast and non-gymnast participants within each age group using independent-sample t-tests. Mean differences (MDs), 95% confidence intervals (CIs), and partial eta-squared (η^2^) effect sizes were reported for the results. A two-way multivariate ANOVA (MANOVA) was performed to evaluate the effects of group (middle school *vs.* university) and activity (gymnast *vs.* non-gymnast) on the combined dependent variables, including spatiotemporal gait parameters (gait speed, cadence, stride length, gait cycle time, and step width) and GRF-X across the gait sub-phases (initial contact, loading response, mid-stance, terminal stance, and pre-swing). Wilks’ lambda (Λ) was used to determine the multivariate significance. When a significant multivariate effect was detected, follow-up two-way ANOVA was conducted for each dependent variable to examine the main effects and Group × Activity interactions. Effect sizes were reported using η^2^. Post-hoc pairwise comparisons were performed using the Bonferroni correction, where appropriate. Statistical significance was set at p < 0.05. Furthermore, a simple linear regression analysis was performed to investigate the relationship between age as the independent variable and spatiotemporal gait parameters (namely, cadence, gait speed, stride length, gait cycle time, and step width) within the gymnast and non-gymnast groups separately.

## 3 Results

### 3.1 Demographic characteristics of the study groups

Demographic data were collected for the selected cases (Table 1). The sample comprised 40 volunteers divided into four groups: 10 non-gymnast female middle school students, 10 gymnast female middle school students, 10 non-gymnast female university students, and 10 gymnast female university students. There were no significant differences in mean age, weight, height, or body mass index among female individuals within the same age group.

**TABLE 1:** Demographic characteristics of the selected cases.

Variable	Middle school female students					Female university students				
	Non-gymnast	Gymnast	MD (95% CI)	*p*	Effect size (η^2^)	Non-gymnast	Gymnast	MD (95% CI)	*p*	Effect size (η^2^)
Age (year)	12.30 ± 0.42	12.60 ± 0.31	0.30 (−0.80, 1.40)	0.57	0.02	21.20 ± 0.51	21.60 ± 0.37	−0.40 (−1.73, 0.93)	0.54	0.02
Weight (kg)	56.80 ± 1.50	54.40 ± 1.82	−2.40 (−7.35, 2.56)	0.32	0.05	55.70 ± 2.35	53.50 ± 2.00	−2.20 (−8.67, 4.27)	0.48	0.03
Height (m)	1.56 ± 0.04	1.48 ± 0.02	−0.07 (−0.17, 0.02)	0.10	0.14	1.56 ± 0.01	1.55 ± 0.01	−0.01 (−0.05, 0.04)	0.74	0.01
BMI (kg/m^2^)	23.81 ± 1.39	24.86 ± 1.05	1.05 (−2.61, 4.71)	0.55	0.02	23.09 ± 0.98	22.41 ± 0.92	−0.68 (−3.50, 2.15)	0.62	0.01

−Values are shown as the mean ± SEM.

−Baseline comparisons between gymnast and non-gymnast participants within each age group were performed using independent-samples t-tests.

−BMI, body mass index; MD, mean difference (calculated as gymnast − non-gymnast); CI, confidence interval.

−Statistical significance was set at *p* < 0.05.

−η^2^, partial eta square.

### 3.2 Spatiotemporal gait parameters of the study groups

#### 3.2.1 Descriptive statistics

The descriptive statistics for spatiotemporal gait parameters are presented in Table 2. Across both age groups, the gymnast participants consistently demonstrated higher gait speed, walking cadence, and stride length than the non-gymnast participants. Conversely, gait cycle time and step width were lower in the gymnast participants. These descriptive trends were observed in both female middle school students and female university students, indicating similar patterns of gait adaptation associated with gymnastics training in both groups.

**TABLE 2:** Descriptive statistics and two-way ANOVA results for spatiotemporal gait parameters according to age group and activity.

Variable	Female middle school students		Female university students		Activity F (P)	Group F (P)	Group × activity F (P)	Effect size (η2) for activity
	Non-gymnast	Gymnast	Non-gymnast	Gymnast				
Gait speed (cm/s)	73.27 ± 2.71	124.00 ± 3.40	75.68 ± 3.11	136.00 ± 5.81	197.86 (<0.001***)	3.33 (0.08)	1.47 (0.23)	0.85
Walking cadence (steps/min)	82.94 ± 2.49	114.30 ± 1.00	88.46 ± 2.02	112.90 ± 1.00	254.08 (<0.001***)	1.38 (0.25)	3.90 (0.06)	0.88
Stride length (cm)	97.72 ± 4.12	125.00 ± 3.07	102.49 ± 2.89	128.00 ± 4.67	49.25 (<0.001***)	1.07 (0.31)	0.06 (0.82)	0.58
Gait cycle time (min)	1.50 ± 0.22	1.07 ± 0.02	1.36 ± 0.03	1.07 ± 0.06	52.22 (<0.001***)	1.97 (0.17)	2.15 (0.15)	0.59
Step width (cm)	9.51 ± 0.47	7.07 ± 0.52	9.90 ± 0.76	7.89 ± 0.31	17.01 (<0.001***)	1.24 (0.27)	0.16 (0.69)	0.32

−Values are shown as the mean ± SEM.

−Statistical significance was evaluated using two-way ANOVA, with Group (middle school *vs.* university) and Activity (gymnast *vs*. non-gymnast) as fixed factors.

−Reported F and *p*-values correspond to the main effects of Activity and Group and their interaction. ***p < 0.001 is statistically significant.

−Partial η^2^ is reported as the measure of effect size for the main effect of Activity.

#### 3.2.2 Multivariate analysis

MANOVA was performed to examine the effects of age group (middle school versus university) and activity type (gymnast versus non-gymnast) on spatiotemporal gait parameters. The analysis revealed that the multivariate interaction effect between Group and Activity was not statistically significant (Wilks’ Λ = 0.80, F (5, 32) = 1.61, *p* = 0.19, partial η^2^ = 0.20). Similarly, the multivariate main effect for the group was not significant (Wilks’ Λ = 0.85, F (5, 32) = 1.16, *p* = 0.35, partial η^2^ = 0.15). In contrast, a highly significant multivariate main effect was observed for activity (Wilks’ Λ = 0.09, F (5, 32) = 65.54, *p* < 0.001, and partial η^2^ = 0.91), indicating a large effect size.

#### 3.2.3 Univariate analysis of gait parameters

Follow-up two-way ANOVAs (Table 2) confirmed a significant main effect of activity across all spatiotemporal gait parameters: gait speed, F (1, 36) = 197.86, p < 0.001, η^2^ = 0.85; walking cadence, F (1, 36) = 254.08, *p* < 0.001, η^2^ = 0.88; stride length, F (1, 36) = 49.25, *p* < 0.001, η^2^ = 0.58; gait cycle, F (1, 36) = 52.22, *p* < 0.001, η^2^ = 0.59; and step width, F (1, 36) = 17.01, *p* < 0.001, η^2^ = 0.32. No significant main effect of group or interaction effect was detected for any variable, as all *p*-values were >0.05.

#### 3.2.4 Bonferroni-adjusted pairwise comparisons for activity

Bonferroni-adjusted pairwise comparisons (Table 3) indicated that gymnast participants had greater gait speed (MD = 55.53, 95% CI (47.52, 63.53)), walking cadence (MD = 27.90, 95% CI (24.35, 31.45)), and stride length (MD = 26.40, 95% CI (18.77, 34.02)), as well as reduced gait cycle duration (MD = −0.36, 95% CI (−0.46, −0.26)) and step width (MD = −2.23, 95% CI (−3.32, −1.13)), compared with non-gymnast participants (all *p* < 0.001). Comparisons within the gymnast group showed no statistically significant differences between middle school and university participants, with small effect sizes (η ≤ 0.15). Because neither the main effect of Group nor the Group × Activity interaction was statistically significant, no additional *post hoc* comparisons between middle school and university participants were performed. These results suggest that activity level, rather than age group, predominantly influences spatiotemporal gait performance.

**TABLE 3:** Bonferroni-adjusted pairwise comparisons for the main effect of activity on spatiotemporal gait parameters.

Variables	Non-gymnast	Gymnast	MD (95%CI)	*P* value
Gait speed (cm/s)	74.47 ± 2.79	130.00 ± 2.79	55.53 (47.52.63.53)	<0.001^***^
Walking cadence (steps/min)	85.70 ± 1.24	113.06 ± 1.24	27.90 (24.35.31.45)	<0.001^***^
Stride length (cm)	100.11 ± 2.66	126.50 ± 2.66	26.40 (18.77.34.02)	<0.001^***^
Gait cycle time (min)	1.43 ± 0.04	1.07 ± 0.04	−0.36 (-0.46 to-0.26)	<0.001^***^
Step width (cm)	9.71 ± 0.38	7.48 ± 0.38	−2.23 (-3.32 to-1.13)	<0.001^***^

−Values are shown as the mean ± SEM.

−Mean differences and 95% CIs were obtained from Bonferroni-adjusted pairwise comparisons following the two-way ANOVA.

−MD, mean difference (calculated as gymnast − non-gymnast); CI, confidence interval.

− ***p < 0.001 is statistically significant.

#### 3.2.5 Linear regression analysis between age and activity

Figure 1 shows the linear regression analysis examining the relationship between age and spatiotemporal parameters in female non-gymnasts. The analysis indicated a weak positive correlation between age and gait speed, stride length, and step width, with correlation coefficients of r = 0.20, r = 0.24, and r = 0.15, respectively. As shown in Figures 1A,C,E, age accounted for increases of 4%, 5.7%, and 2.2% in gait speed, stride length, and step width, respectively. Additionally, an intermediate positive correlation (r = 0.40) was observed between age and walking cadence, with age explaining a 16% increase in cadence (Figure 1B). However, a significant intermediate negative correlation (r = −0.47) was observed between age and gait cycle time, with age accounting for a 22.3% decrease in gait cycle time, as shown in Figure 1D.

**FIGURE 1:**
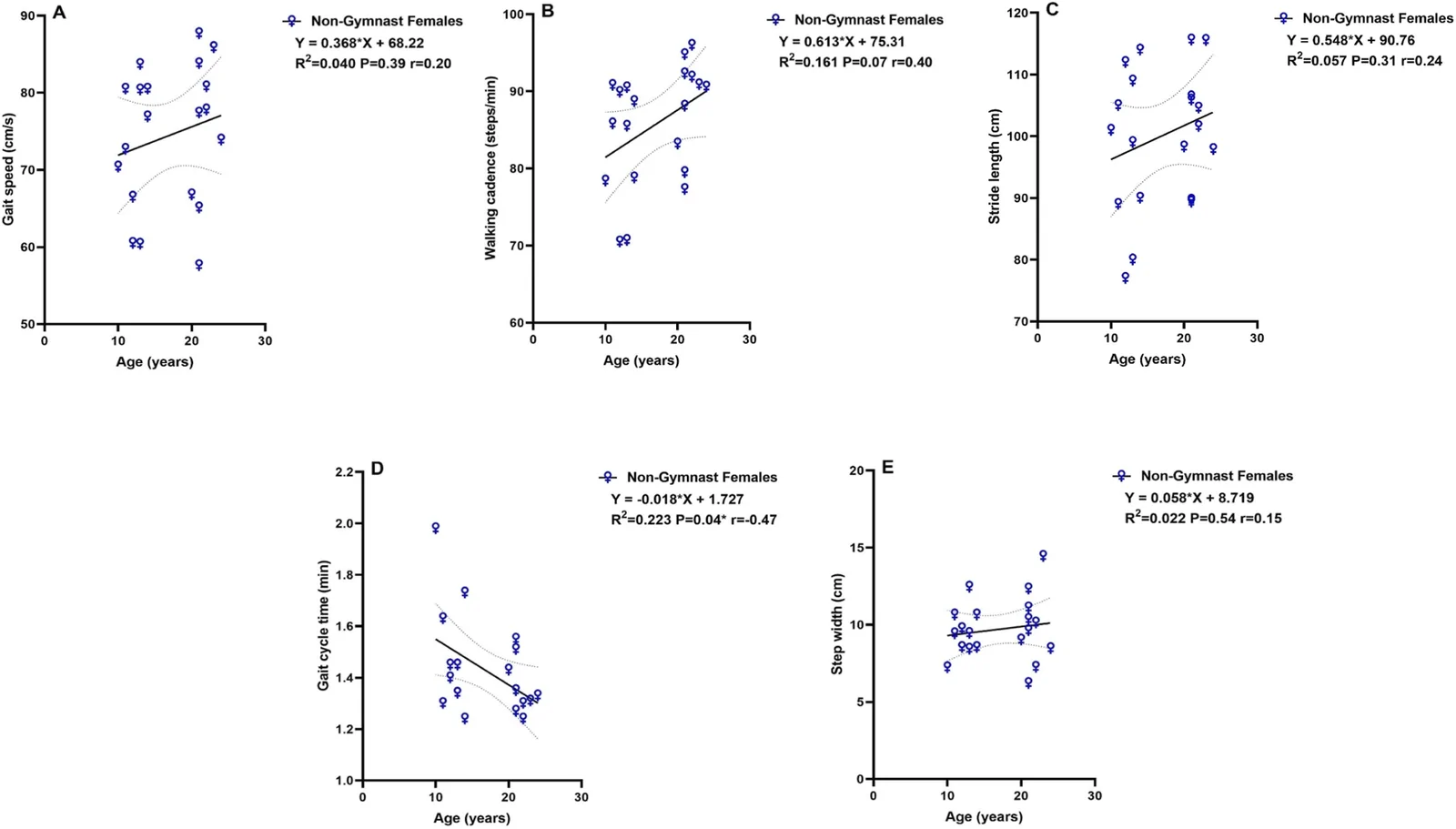
Linear regression of age (years) *vs.* spatiotemporal gait parameters in female non-gymnasts. **(A)** Gait speed. **(B)** Walking cadence. **(C)** Stride length. **(D)** Gait cycle time. **(E)** Step width.

Figure 2 shows the linear regression analysis between age and spatiotemporal parameters in female gymnasts. There was a weak positive correlation between age and stride length, gait cycle time, and step width (r = 0.21, r = 0.11, and r = 0.26, respectively). As indicated in Figures 2C–E, an increase in age explained 4.3%, 1.3%, and 6.5% increases in stride length, gait cycle time, and step width, respectively. There was an intermediate positive correlation (r = 0.42) between age and gait speed, with an increase in age explaining an 18.1% increase in gait speed (Figure 2A). A weak negative correlation (r = −0.21) was observed between age and walking cadence, indicating that an increase in age accounted for a 4.6% decrease in walking cadence (Figure 2B).

**FIGURE 2:**
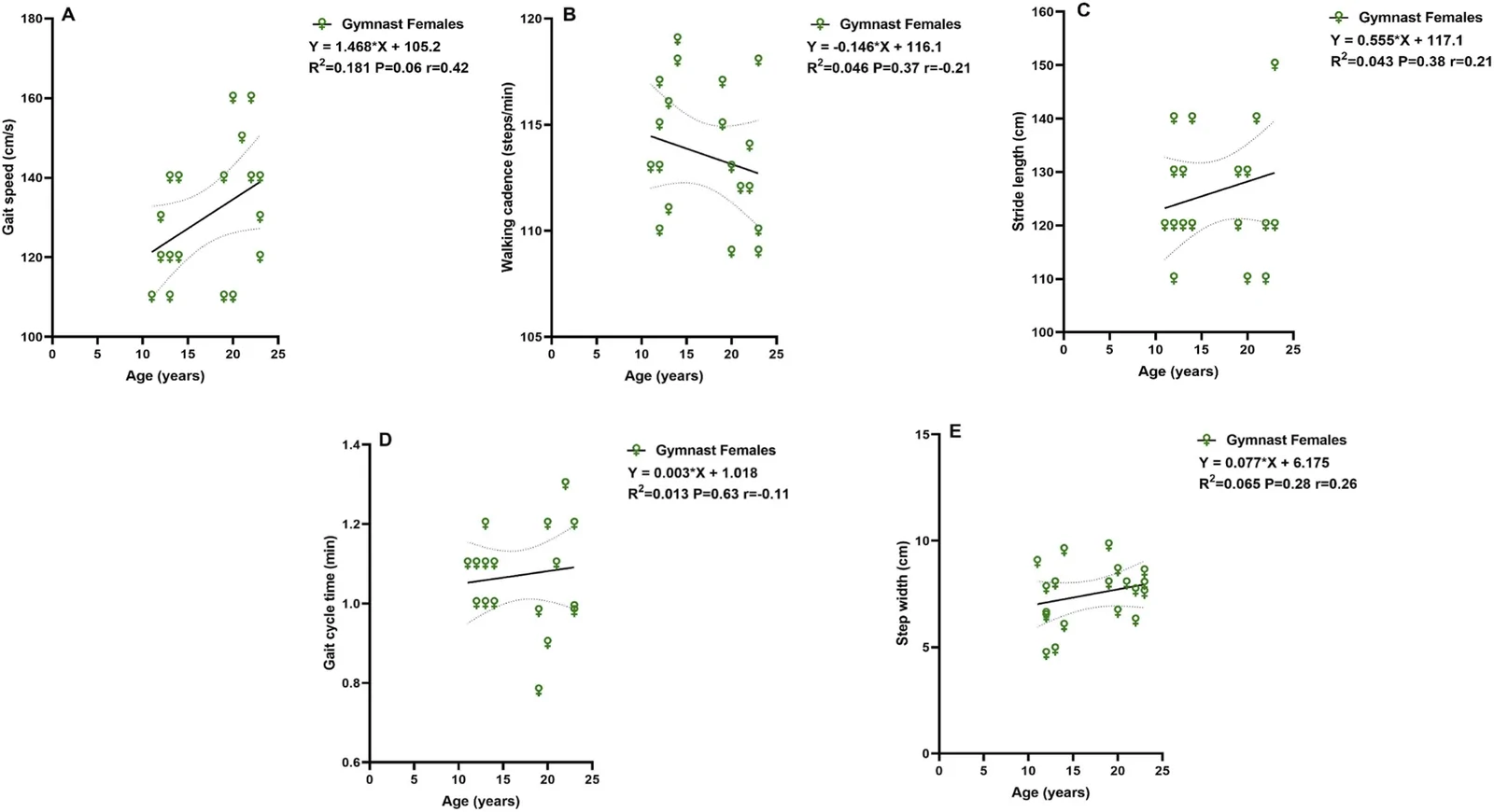
Linear regression of age (years) *vs.* spatiotemporal gait parameters in female gymnasts. **(A)** Gait speed. **(B)** Walking cadence. **(C)** Stride length. **(D)** Gait cycle time. **(E)** Step width.

### 3.3 Kinetics anteroposterior ground reaction force in the study groups

#### 3.3.1 Descriptive statistics

The descriptive statistics for the GRF-X parameters are presented in Table 4 and Figure 3. Across both age groups, gymnast participants exhibited more negative GRF-X values during the initial contact, loading response, and mid-stance phases compared with non-gymnast participants. In contrast, the terminal stance and pre-swing GRF-X values were comparable between gymnast and non-gymnast participants in both female middle school students and female university students.

**TABLE 4:** Descriptive statistics and two-way ANOVA results for anteroposterior ground reaction force (N/BW) parameters according to age group and activity.

Variable	Female middle school students		Female university students		Activity F (*p*)	Group F (*p*)	Group × activity F (*p*)	Effect size (η2) for activity
	Non-gymnast	Gymnast	Non-gymnast	Gymnast				
Kinetics anteroposterior GRF (N/BW)								
-Initial contact phase	−1.39 ± 0.15	−3.43 ± 0.50	−1.98 ± 0.45	−3.70 ± 0.43	21.34 (<0.001***)	1.14 (0.29)	0.14 (0.71)	0.37
-Loading response phase	−6.22 ± 0.52	−9.98 ± 0.61	−5.32 ± 1.60	−6.85 ± 2.19	3.50 (0.07)	2.04 (0.16)	0.62 (0.44)	0.09
-Mid-stance phase	0.46 ± 0.08	−0.95 ± 0.13	0.53 ± 0.12	−1.01 ± 0.07	199.65 (<0.001***)	0.01 (0.94)	0.40 (0.53)	0.85
-Terminal-stance phase	11.46 ± 0.67	12.56 ± 1.00	11.88 ± 0.75	12.02 ± 0.92	0.53 (0.47)	0.004 (0.95)	0.33 (0.57)	0.01
-Pre-swing phase	1.99 ± 0.20	2.36 ± 0.29	2.18 ± 0.25	1.94 ± 0.22	0.08 (0.77)	0.22 (0.65)	1.61 (0.21)	0.002

−Values are shown as the mean ± SEM.

−Statistical significance was evaluated using two-way ANOVA, with Group (middle school *vs.* university) and Activity (gymnast *vs.* non-gymnast) as fixed factors.

−Reported F and *p*-values correspond to the main effects of Activity and Group and their interaction. ***p < 0.001 is statistically significant.

−Partial η^2^ is reported as the measure of effect size for the main effect of activity.

**FIGURE 3:**
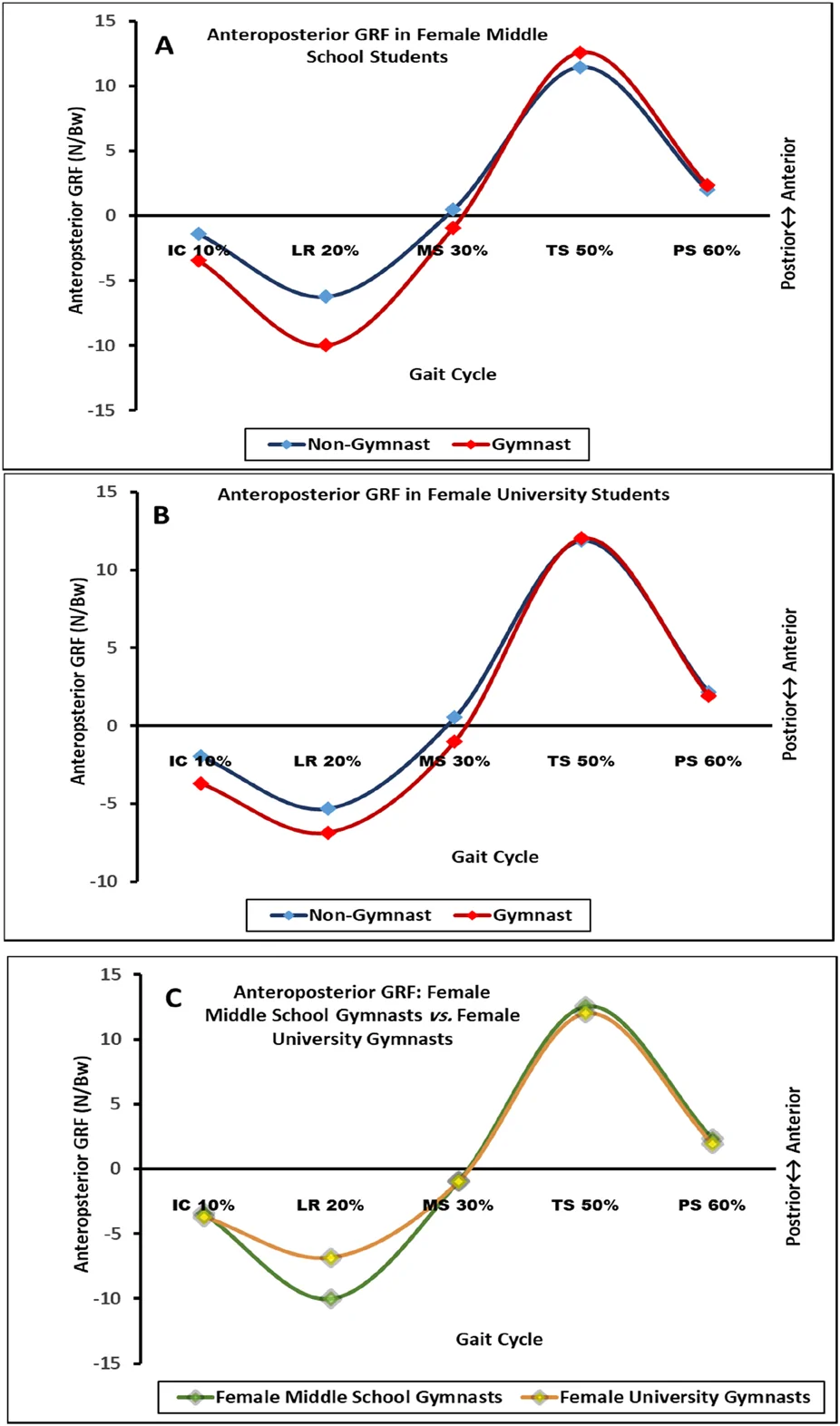
Kinetics of anteroposterior ground reaction force (GRF-X) (N/BW) as a function of normalized gait cycle (0–100%) in the study groups. **(A)** Comparison between non-gymnast and gymnast female middle school students. **(B)** Comparison between non-gymnast and gymnast female university students. **(C)** Comparison between female middle school gymnasts and university female gymnasts. IC, initial contact (10%); LR, loading response (20%); MS, mid-stance (30%); TS, terminal stance (50%); PS, pre-swing (60%).

#### 3.3.2 Multivariate analysis

MANOVA revealed a significant multivariate main effect of activity on GRF-X parameters (Wilks’ Λ = 0.09, F (5, 32) = 64.23, *p* < 0.001, η^2^ = 0.91), indicating a large effect size. In contrast, neither the main effect of Group, Wilks’ Λ = 0.90, F (5, 32) = 0.74, *p* = 0.60, η^2^ = 0.10, nor the Group × Activity interaction, Wilks’ Λ = 0.92, F (5, 32) = 0.52, *p* = 0.76, η^2^ = 0.08, reached statistical significance.

#### 3.3.3 Univariate analysis

Follow-up two-way ANOVAs (Table 4) indicated a significant main effect of activity for the initial contact phase, F (1, 36) = 21.34, *p* < 0.001, η^2^ = 0.37, and mid-stance phase, F (1, 36) = 199.65, *p* < 0.001, η^2^ = 0.85, reflecting moderate and large effect sizes, respectively. No significant activity effects were observed for the loading response (F (1, 36) = 3.50, *p* = 0.07), terminal stance (F (1, 36) = 0.53, *p* = 0.47), or pre-swing (F (1, 36) = 0.08, *p* = 0.77) phases. Additionally, neither the main effect of group nor the interaction between Group and Activity was significant across any GRF-X variable (all *p* > 0.05).

#### 3.3.4 Bonferroni-adjusted pairwise comparisons

Bonferroni-adjusted pairwise comparisons (Table 5) revealed that gymnast participants demonstrated significantly more negative initial contact values compared with non-gymnast participants (MD = −1.88, 95% CI (−2.71 to −1.05), p < 0.001). Similarly, significant differences were observed in the mid-stance phase (MD = −1.47, 95% CI (−1.68 to −1.26), *p* < 0.001), indicating altered loading patterns during the stance phase in gymnasts. No significant pairwise differences were detected for the loading response (MD = −2.65, 95% CI −5.51 to 0.22, *p* = 0.07), terminal stance (MD = 0.62, 95% CI −1.10 to 2.33, *p* = 0.47), or pre-swing (MD = 0.07, 95% CI −0.42 to 0.56, *p* = 0.77) phases.

**TABLE 5:** Bonferroni-adjusted pairwise comparisons for the main effect of activity on GRF-X (N/BW) parameters.

Variable	Non-gymnast	Gymnast	MD (95% CI)	*p*-value
Kinetics GRF-X (N/BW)				
-Initial contact phase	−1.69 ± 0.29	−3.57 ± 0.29	−1.88 (−2.71, −1.05)	<0.001^***^
-Loading response phase	−5.77 ± 1.00	−8.42 ± 1.00	−2.65 (−5.51, 0.22)	0.07
-Mid-stance phase	0.49 ± 0.07	−0.98 ± 0.07	−1.47 (−1.68, −1.26)	<0.001^***^
-Terminal stance phase	11.67 ± 0.60	12.29 ± 0.60	0.62 (−1.10, 2.33)	0.47
-Pre-swing phase	2.08 ± 0.17	2.15 ± 0.17	0.07 (−0.42, 0.56)	0.77

−Values are shown as the mean ± SEM.

−Mean differences and 95% CIs were obtained from Bonferroni-adjusted pairwise comparisons following the two-way ANOVA.

−MD: mean difference (calculated as gymnast − non-gymnast); CI, confidence interval; ANOVA, analysis of variance.

−***p < 0.001 is statistically significant.

## 4 Discussion

Recent findings have indicated significant differences in cadence, speed, cycle time, and step width between female middle school gymnasts and their non-gymnast counterparts. Similarly, female university students engaged in gymnastics exhibit notable variations in cadence, speed, and stride length compared with those not involved in the sport. Additionally, average GRF-X values during the initial contact, loading response, and mid-stance phases differed substantially between female middle school gymnasts and non-gymnasts. A pronounced difference was also evident between female gymnasts and non-gymnast university students during the initial contact and mid-stance phases.

The observed alterations in spatiotemporal gait characteristics suggest that long-term gymnastics training improves locomotor efficiency and movement control while walking. The current findings are consistent with those of Tudor-Locke et al. (2019), who identified a positive predictive correlation between cadence and moderate-intensity training exercises, indicating increased gait time in adults. Leacox et al. (2025) also found that when 30 adults ran from a slow to a fast pace, both GRFs and cadence increased with speed. This correlation offers a coherent and logical explanation for the observed variations in spatiotemporal gait parameters between gymnasts and non-gymnasts, particularly during prolonged and rhythmic ambulatory activities.

Notable differences were observed in GRF-X across the different sub-phases of the gait cycle. Age-matched differences among the four groups likely also played a significant role in the GRF variations observed in the current study. This is consistent with the findings of Ancillao et al. (2018), who demonstrated that GRF, power, and moment can be reliably measured and detected using kinematic gait analysis in various sports activities, including walking, running, and jumping. Recent studies have also highlighted the impact of gender and age on kinetic variables in sports biomechanics, revealing significant variations in GRF between male and female athletes, even when age and activity level were controlled for. Additionally, age-related differences in neuromuscular control influence GRF and other kinetic parameters in high-impact sports activities.

In the same context, prolonged gymnast training modifies dynamic joint stabilization and lower limb stiffness, which directly influences the anteroposterior GRF-X observed during the loading response and mid-stance phases. Silva et al. (2025) suggested that enhanced adaptation of the lower limbs reduces the impact of GRFs, leading to a less painful and more stable landing. Furthermore, they confirmed that the impact of GRF was more pronounced during the landing phase among female gymnasts, who subject their lower limb joints to higher injury risks. Coaches focus on this aspect during training sessions with athletes (Silva et al., 2025).

Focusing on power propulsion, Di Giminiani et al. (2020) stated that the co-activation of the thigh muscles explains leg stiffness adjustments at different drop heights. In contrast, the co-activation of the shank muscles is mostly responsible for power propulsion during the pre-contact and braking phases. Moeskops et al. (2024) investigated the impact of neuromuscular training on muscle architecture, isometric force production, and stretch-shortening cycle function in three groups of trained young female gymnasts, focusing on concepts such as neuromuscular control, the stiffness regulation of braking propulsion mechanics, and the kinetic chain. Their findings indicated that relative peak force, drop jump kinetics, and gastrocnemius muscle thickness, which are critical in the pre-swing phase of the gait cycle, improved in the gymnast group undergoing neuromuscular training compared with the control group or gymnast group only. In addition, Almansoof et al. (2023) highlighted the critical role of the core region, specifically the lumbopelvic–hip complex, as the central link within the kinetic chain during most athletic activities. This region plays a key role in efficiently transmitting forces between the upper and lower limbs. The proper functioning of the kinetic chain relies on the integration of neuromuscular, sensorimotor, and neurocognitive systems. Disruptions at any point in this chain may lead to compensatory movement patterns, increased mechanical stress on the distal segments, and a higher risk of overuse injuries. Therefore, adopting a comprehensive approach that assesses the integrity of the kinetic chain is essential for optimizing athletic performance and reducing injury risk.

The biomechanical adaptations observed during walking are consistent with previous evidence demonstrating that gymnastics-specific jumping and landing tasks improve balance control, proprioception, and force regulation in healthy individuals. Kyselovičová and Zemková (2010) studied the effect of maximal jumps and sport-specific exercises on the movement of the center of gravity, which is the point through which the GRF passes during vertical and frontal splits. Their study, which included eight aerobic gymnasts, identified significant differences in the medio-lateral and anteroposterior directions of the center of gravity before and after exercise. The findings suggest that post-exercise balance disturbances are affected by both the intensity of proprioceptive stimulation and the type of exercise performed. This highlights the significance of exercise-specific effects on balance and gait. Glynn et al. (2022) further supported this biomechanical perspective. Their study demonstrated inter-limb asymmetry in landing force among injured gymnasts, and uninjured gymnasts also exhibited asymmetry in the mean value of the peak vertical GRF. However, the likelihood of an ankle injury could not be determined based on the magnitude of the landing force or inter-limb asymmetries. This finding highlights the potential of our data to predict gymnast injuries based on variations in gait and GRF. Colclough et al. (2018) confirmed that a jump training program improved female gymnasts’ frontal plane projection angles and recommended incorporating it into warm-ups and training regimens to mitigate the risk factors for knee injuries.

Age-related maturation is another important factor that may contribute to the differences in gait mechanics and GRFs observed between the adolescent (middle school) and university groups in this study. Bach et al. (2021) highlighted the clinical significance of maturing normal gait patterns, particularly in the double support and swing phases of the gait cycle, among children aged 2–9 years and healthy young adults. They found variations in the center of mass among these age categories, as GRFs intersect above the center of mass during single support, as reflected in the mean values in the horizontal and vertical directions (Vielemeyer et al., 2021). These findings align with the variations in mean GRF-X values observed in the current study. These observations validate the significant results of this study, which revealed distinct gait characteristics among the four groups representing different age categories during normal walking.

Several studies have explored the impact of training exercises at different intervals on the symmetrical strength of the lower limbs in young female gymnasts. Ferri-Caruana et al. (2020) and Bogdanis et al. (2019) demonstrated that resistance training exercises in gymnasts improved their jumping performance and walking speed. These findings underscore the positive influence of structured training programs on motor skills and physical performance in children. Furthermore, Colclough et al. (2018) showed that 1 month of training significantly improved the frontal plane projection angle in young female gymnasts, reducing the risk of knee injuries during rehabilitation. Similar to our study, Hassan et al. (2022) investigated the relationship between ankle dorsiflexion range of motion, frontal plane projection angle, and patellofemoral pain syndrome. Their results revealed that a decreased dorsiflexion angle may reduce the base of support, which reflects the step width and limits the frontal plane projection angle during functional activities, thereby increasing the likelihood of injuries. Čeklić and Šarabon (2021) and Šarabon et al. (2021) further supported these findings by attributing differences in joint function among gymnasts to the overload injuries sustained during training. They also noted that factors such as balance, strength, and asymmetries of the body contribute to these variations. Ferri-Caruana et al. (2020) and Exell et al. (2015) also demonstrated improvements in jumping, sports skills, and the ability to change direction or speed through a training intervention program that had a direct impact on these factors during walking. Variations in GRF may also be influenced by these power measurements.

Biomechanical evaluation of injury prevention strategies in gymnasts is crucial for improving athlete performance and safety. Gymnasts are frequently subjected to high-impact forces, which can result in acute trauma and chronic overuse injuries. This study investigates how biomechanical concepts related to barefoot walking, such as force analysis and joint kinematics, can be applied to identify and mitigate gymnast injury risks. According to Lubis et al. (2025), sports scientists and coaches have access to cutting-edge tools such as motion capture systems, force plates, and electromyography, which can be used to examine a person’s movement patterns and identify areas of susceptibility. Their findings highlight the importance of optimal landing techniques, improving flexibility, building muscle, and using proper safety equipment to prevent injuries. Integrating biomechanical evaluations into training regimens constitutes a highly effective strategy for reducing injury rates, enhancing performance, and promoting a safer environment for competitive gymnasts. This strategy underscores the importance of biomechanics in enhancing the efficacy of injury prevention techniques and supporting long-term sports health. Furthermore, biomechanical data can inform the development of orthotic devices that stabilize joints and decrease the risk of overuse injuries, as well as protective equipment such as padded mats. By consistently incorporating biomechanical evaluations into training regimens, the safety of practice and competition for gymnasts can be improved, thereby reducing the injury rate.

Our study indicated that age was positively correlated with gait speed, walking cadence, stride length, and step width but negatively correlated with gait cycle time in female non-gymnasts. In addition, a positive correlation was observed between age and gait speed, stride length, gait cycle time, and step width, but a negative correlation was found with walking cadence in female gymnasts. While some gait kinematics appear to reach an adult-like pattern by the age of 7 or 8 years, gait parameters, such as step length, cadence, single- and double-limb support times, and trunk motion smoothness, continue to mature during adolescence (Froehle et al., 2013; Leban et al., 2019). During this developmental period, individual body segments undergo absolute and relative changes in size, which may affect the effectiveness and efficiency of gait stabilization strategies (Standing et al., 2019). These biomechanical adaptations may explain the positive correlations observed between age and parameters associated with locomotor efficiency, such as stride length and gait speed, as older participants generally exhibit greater lower limb length, improved neuromuscular coordination, and more efficient propulsion while walking.

Conversely, the negative correlations observed between age and cadence or step width may reflect the progressive maturation of postural control and dynamic balance. As gait becomes more stable with age and training experience, fewer steps are required to maintain walking speed, resulting in a lower cadence, whereas a narrower base of support indicates improved mediolateral stability and movement efficiency. The results of Petran et al. (2023) support the current findings, as they indicated that maintaining balance is connected with increasing step width. In two groups of young female gymnasts, G1 (6–8 years) and G2 (9–10 years), the impact of anthropometric indices on balance metrics was assessed. According to their findings, there is a positive link between step and age as one of the anthropometric variables. Furthermore, Opala-Berdzik et al. (2021) reported that the anteroposterior center of foot pressure mean velocity in artistic gymnasts was inversely associated with training experience, age, body height, body mass, and maturity offset, suggesting that increasing age and training contribute to enhanced postural stability and more efficient gait patterns. Collectively, these findings indicate that the positive and negative age-related correlations observed in the present study are likely attributable to the combined effects of growth, neuromuscular maturation, and long-term gymnastics training on the biomechanics of gait.

Various factors may influence the development of postural control, including age (Barela et al., 2003; Peterson et al., 2006; Cuisinier et al., 2011) and an individual’s experiential practice in sports, that requires significant balance control (Busquets et al., 2018). Postural stability during ambulation is a critical element that mitigates the risk of falling. Researchers have identified optimal methods to aid individuals whose postural stability deficits are mostly associated with gait metrics. Spatiotemporal characteristics are commonly used to ascertain deviations from the norm in certain populations and serve as accessible indicators for examining the effects of aging on gait patterns (Al-Makhalas et al., 2023). Age, as an indicator of experience, enhances reweighting mechanisms associated with postural regulation. The assimilation of sensory information may not attain adult levels until the ages of 12 to 15 years, even with continuous manipulation of sensory inputs (Busquets et al., 2018).

The gait and kinetic outcomes of gymnasts can be significantly affected by potential confounders, including training volume, intensity, and skill level of the gymnast. Thus, Taneja et al. (2023) investigated how exercise training affects gymnasts’ physical fitness metrics. The researchers indicated that the gymnast experienced improvements in lower limb strength and trunk endurance because of most of the physical training activities. Force plate inertial measuring devices were used to assess biomechanical stress during training sessions in female artistic gymnasts. The results indicated that the biomechanical stress experienced by gymnasts is influenced by variations in skill. Consequently, the management of training schedules and the development of injury prevention strategies necessitate a comprehensive understanding of the stressors encountered by gymnasts (Boylen et al., 2025). In addition, the highest- and lowest-intensity training sessions of nine gymnasts were examined by Batista et al. (2018). Their analysis revealed that these sessions primarily involved low-intensity exercises, including flexibility training and apparatus-handling techniques. Training sessions also included pauses for explanations and corrections to enhance technique, tactics, physical fitness, intellectual growth, and psychological development, all of which ultimately contribute to improved performance in gymnasts.

The conceptual contribution across the two age groups in the current study was further supported by Steinberg et al. (2025). This study examined the physiological characteristics of young female gymnasts across artistic, acrobatic, and rhythmic disciplines during childhood and adolescence. They hypothesized that these physiological traits would differ according to gymnast discipline and age group. Their findings indicated that artistic gymnasts were shorter than their acrobatic and rhythmic counterparts, and they exhibited lower bone strength than rhythmic and acrobatic gymnasts, although they shared similar body mass index, body fat, age patterns, and training volume histories. This combination of factors may elevate the risk of musculoskeletal issues when coupled with intense, high-impact training. Therefore, the study’s findings could aid medical teams and sports trainers in establishing “norms” for different age groups based on anticipated developments during early and late maturation. This knowledge can inform the optimization, customization, and modification of training programs for athletes in the future.

To complement the factorial two-way ANOVA, exploratory linear regression analysis was performed to examine whether age was linearly associated with spatiotemporal gait parameters across the study sample. Although age was primarily analyzed as a categorical factor within the factorial design, regression analysis was conducted to explore potential age-related trends in gait characteristics and to generate preliminary evidence that may inform future longitudinal investigations involving a continuous age distribution.

However, the regression findings should be interpreted with caution. The age variable consisted of two distinct, non-continuous cohorts (11–14 and 20–24 years) rather than a continuous distribution. This clustered age structure may violate the assumptions of linear regression and introduce bias when estimating age-related associations in the data. Therefore, the regression analysis should be regarded as exploratory; the factorial two-way ANOVA, which appropriately reflects the categorical study design, provides the primary basis for interpreting the findings.

The present study has several limitations. First, the sample size was relatively small. Second, only female participants were included because female individuals are reported to be at greater risk of gymnastics-related injuries, particularly at a younger age. Consequently, the findings cannot be generalized to male athletes alone. Finally, the clustered age distribution limited the interpretation of age-related trends identified using exploratory regression analysis. Future studies should include larger samples, both genders, a broader and more continuous age range, and longitudinal follow-up to better characterize the influence of age and gymnastics training on gait biomechanics and GRF characteristics of the lower limbs.

## 5 Conclusion

The current study revealed that gymnastics training induces significant changes in spatiotemporal gait characteristics and anteroposterior GRF-X when comparing female gymnasts with their non-gymnast counterparts of the same age. This highlights the necessity of examining differences between athletic and non-athletic groups at various developmental stages to comprehend the influence of sports participation, athletic status, and age on gait. Practically, these findings can inform the design of training, rehabilitation, and injury prevention programs tailored to the specific needs of female athletes and non-athletes at varying developmental ages. Analyzing gait characteristics can aid clinicians, physiotherapists, and sports experts in identifying movement patterns that may affect injury risk and athletic performance. Rehabilitation strategies may benefit from focusing on enhancing lower limb muscle strength, optimizing foot clearance and foot–ground contact, and improving dynamic stability during walking and sports-specific activities. Additionally, strategies that emphasize controlling step width and lower limb stability could be particularly crucial for gymnasts who frequently experience high mechanical loads and challenging balance tasks.

However, it is essential to interpret these findings with caution because of limitations such as a relatively small sample size, exclusive focus on female participants, and clustered age distribution. Therefore, further research involving a larger sample, including both genders and a wider age range, is necessary to verify these results and establish normative reference data for gait and GRF characteristics in athletic populations.

## Data Availability

The original contributions presented in the study are included in the article/supplementary material; further inquiries can be directed to the corresponding authors.

## References

[B1] Al-Makhalas A. Abualait T. Ahsan M. Abdulaziz S. Al Muslem W. (2023). A gender based comparison and correlation of spatiotemporal gait parameters and postural stability. Acta Bio Medica Atenei Parm. 94 (2), e2023057. 10.23750/abm.v94i2.13602 PMC10210557 37092642

[B2] Almansoof H. S. Nuhmani S. Muaidi Q. (2023). Role of ankle dorsiflexion in sports performance and injury risk: a narrative review. Electron. J. General Med. 20 (5), em521. 10.29333/ejgm/13412 PMC10893580 38406779

[B3] Ancillao A. Tedesco S. Barton J. O’flynn B. (2018). Indirect measurement of ground reaction forces and moments by means of wearable inertial sensors: a systematic review. Sensors 18 (8), 2564. 10.3390/s18082564 30081607 PMC6111315

[B4] Asseman F. B. Caron O. Crémieux J. (2008). Are there specific conditions for which expertise in gymnastics could have an effect on postural control and performance? Gait & Posture 27 (1), 76–81. 10.1016/j.gaitpost.2007.01.004 17337190

[B5] Bach M. M. Daffertshofer A. Dominici N. (2021). The development of mature gait patterns in children during walking and running. Eur. J. Appl. Physiology 121 (4), 1073–1085. 10.1007/s00421-020-04592-2 PMC7966230 33439307

[B6] Barela J. A. Jeka J. J. Clark J. E. (2003). Postural control in children. Exp. Brain Res. 150 (4), 434–442. 10.1007/s00221-003-1441-5 12739087

[B7] Batista A. Gomes T. N. Garganta R. Ávila-Carvalho L. (2018). Training intensity of group in rhythmic gymnastics. Ovidius Univ. Ann. Ser. Phys. Educ. Sport/Sci. Mov. Health 18 (1), 17–24.

[B8] Błażkiewicz M. Kępczyński A. Wit A. (2019). Comparative analysis of kinetics parameters during different landing after split front leaps. Pol. J. Sport Tour. 26 (2), 3–6. 10.2478/pjst-2019-0007

[B9] Bobo-Arce M. Méndez-Rial B. (2013). Determinants of competitive performance in rhythmic gymnastics. A review. J. Hum. Sport Exerc. 8 (Proc3), 711–727. 10.4100/jhse.2013.8.proc3.18

[B10] Bogdanis G. Donti O. Papia A. Donti A. Apostolidis N. Sands W. (2019). Effect of plyometric training on jumping, sprinting and change of direction speed in child female athletes. Sports 7 (5), 116. 10.3390/sports7050116 31108905 PMC6572020

[B11] Boylen A. M. Campbell R. A. Drinkwater E. J. Bradshaw E. J. (2025). Current biomechanical methods and practices of physical training load monitoring in women’s artistic gymnastics: a scoping review. J. Sports Sci. 43 (18), 1992–2006. 10.1080/02640414.2025.2528442 40607678

[B12] Budiarti R. Siswantoyo S. Sukamti E. R. (2022). “ *Explosive Power and Muscle Flexibility in Junior Gymnasts of Aerobic Gymnastic Based on Different Sexes,” in* Proceedings of the Conference on Interdisciplinary Approach in Sports in conjunction with the 4th Yogyakarta International Seminar on Health, Physical Education, and Sport Science (COIS-YISHPESS 2021). (Paris: Atlantis Press), 144–147. 10.2991/ahsr.k.220106.027

[B13] Busquets A. Aranda-Garcia S. Ferrer-Uris B. Marina M. Angulo-Barroso R. (2018). Age and gymnastic experience effects on sensory reweighting processes during quiet stand. Gait & Posture 63, 177–183. 10.1016/j.gaitpost.2018.05.009 29763813

[B14] Carrick F. R. Oggero E. Pagnacco G. Brock J. B. Arikan T. (2007). Posturographic testing and motor learning predictability in gymnasts. Disabil. Rehabilitation 29 (24), 1881–1889. 10.1080/09638280601141335 17852265

[B15] Čeklić U. Šarabon N. (2021). Comparison between gymnasts and non-gymnasts in isometric strength of the lower limbs. Eur. J. Transl. Myology 31 (1), 9663. 10.4081/ejtm.2021.9663 PMC8056165 33709654

[B16] Cerasela D. S. Andrei D. Nicoleta M. S. Petru D. M. Silviu B. I. Adrian N. (2020). “ *Kinematic Quantification of Knee Joint Asymmetry During Preparatory Phase of a Standing Backward Tucked Salto, ” In* 2020 IEEE 18th International Symposium on Intelligent Systems and Informatics (SISY) *(Subotica, Serbia: IEEE)* . 10.1109/SISY50555.2020.9217081

[B17] Cicchella A. (2009). Kinematics analysis of selected rhytmic gymnastic leaps. J. Hum. Sport Exerc. 4 (1), 40–47. 10.4100/jhse.2009.41.05

[B18] Colclough A. Munro A. G. Herrington L. C. Mcmahon J. J. Comfort P. (2018). The effects of a four week jump-training program on frontal plane projection angle in female gymnasts. Phys. Ther. Sport 30, 29–33. 10.1016/j.ptsp.2017.11.003 29366914

[B19] Coppola S. Albano D. Sivoccia I. Vastola R. (2020). Biomechanical analysis of a rhythmic gymnastics jump performed using two run-up techniques. J. Phys. Educ. Sport 20 (1), 37–42. 10.7752/jpes.2020.01005

[B20] Croix G. Chollet D. Thouvarecq R. (2010). Effect of expertise level on the perceptual characteristics of gymnasts. J. Strength & Cond. Res. 24 (6), 1458–1463. 10.1519/jsc.0b013e3181d2c216 20453683

[B21] Cuisinier R. Olivier I. Vaugoyeau M. Nougier V. Assaiante C. (2011). Reweighting of sensory inputs to control quiet standing in children from 7 to 11 and in adults. PLoS ONE 6 (5), e19697. 10.1371/journal.pone.0019697 21573028 PMC3090421

[B22] Dallas G. Theodorou A. S. (2018). The influence of a hurdle target point on the kinematics of the handspring vault approach run during training. Sports Biomech. 19 (4), 467–482. 10.1080/14763141.2018.1497196 30136894

[B23] Di Giminiani R. Giovannelli A. Capuano L. Izzicupo P. Di Blasio A. Masedu F. (2020). Neuromuscular strategies in stretch–shortening exercises with increasing drop heights: the role of muscle coactivation in leg stiffness and power propulsion. Int. J. Environ. Res. Public Health 17 (22), 8647. 10.3390/ijerph17228647 33233323 PMC7700220

[B24] Elhafez S. M. Ashour A. A. Elhafez N. M. Elhafez G. M. Abdelmohsen A. M. (2019). Percentage contribution of lower limb moments to vertical ground reaction force in normal gait. J. Chiropr. Med. 18 (2), 90–96. 10.1016/j.jcm.2018.11.003 31367195 PMC6656910

[B25] Exell T. A. Robinson G. Irwin G. (2015). Asymmetry analysis of the arm segments during forward handspring on floor. Eur. J. Sport Sci. 16 (5), 545–552. 10.1080/17461391.2015.1115558 26625144

[B26] Ferri-Caruana A. Roig-Ballester N. Romagnoli M. (2020). Effect of dynamic range of motion and static stretching techniques on flexibility, strength and jump performance in female gymnasts. Sci. Gymnast. J. 12 (1), 87–100. 10.52165/sgj.12.1.87-100

[B27] Froehle A. W. Nahhas R. W. Sherwood R. J. Duren D. L. (2013). Age-related changes in spatiotemporal characteristics of gait accompany ongoing lower limb linear growth in late childhood and early adolescence. Gait & Posture 38 (1), 14–19. 10.1016/j.gaitpost.2012.10.005 23159678 PMC3580126

[B28] Gateva M. (2019). Modified field test for determining the specific endurance in rhythmic gymnastics. J. Appl. Sports Sci. 3 (1), 3–12. 10.37393/jass.2019.01.1

[B146] Glynn B. Laird J. Herrington L. Rushton A. Heneghan N. R. (2022). Analysis of landing performance and ankle injury in elite British artistic gymnastics using a modified drop land task: a longitudinal observational study. Phy. Ther. Sport 55, 61–69. 10.1016/j.ptsp.2022.01.006 35272101

[B29] Gogoi H. Rajpoot Y. S. Borah P. (2021). A prospective cohort study to predict running-related lower limb sports injuries using gait kinematic parameters. Phys. Educ. Theory Methodol. 21 (1), 69–76. 10.17309/tmfv.2021.1.09

[B30] Gómez-Landero L. A. Leal Del Ojo P. Walker C. Floría P. (2021). Static balance performance differs depending on the test, age and specific role played in acrobatic gymnastics. Gait & Posture 90, 48–54. 10.1016/j.gaitpost.2021.07.023 34390922

[B31] Hall E. Bishop D. C. Gee T. I. (2016). Effect of plyometric training on handspring vault performance and functional power in youth female gymnasts. PLOS ONE 11 (2), e0148790. 10.1371/journal.pone.0148790 26859381 PMC4747498

[B32] Hassan K. A. Youssef R. S. E.-E. Mahmoud N. F. Eltagy H. El-Desouky M. A. (2022). The relationship between ankle dorsiflexion range of motion, frontal plane projection angle, and patellofemoral pain syndrome. Foot Ankle Surg. 28 (8), 1427–1432. 10.1016/j.fas.2022.08.003 35989174

[B33] Hume P. A. Hopkins W. G. Robinson D. M. Robinson S. Hollings S. (1993). Predictors of attainment in rhythmic sportive gymnastics. J. Sports Medicine Physical Fitness 33 (4), 367–377. 8035585

[B34] Kyselovičová O. Zemková E. (2010). Postural stability in aerobic gymnastics specific positions. Sport Sci. Rev. 19 (3-4), 109–116. 10.2478/v10237-011-0020-0

[B35] Lamoth C. J. C. Van Lummel R. C. Beek P. J. (2009). Athletic skill level is reflected in body sway: a test case for accelometry in combination with stochastic dynamics. Gait & Posture 29 (4), 546–551. 10.1016/j.gaitpost.2008.12.006 19138522

[B36] Leacox A. Fashingbauer L. Ferguson T. Zajakowski A. Baum B. Reinking M. (2025). The effect of running speed on cadence and running kinetics. Int. J. Sports Phys. Ther. 20 (7), 957–963. 10.26603/001c.140544 PMC12222555 40620407

[B37] Leban B. Cimolin V. Porta M. Arippa F. Pilloni G. Galli M. (2019). Age-related changes in smoothness of gait of healthy children and early adolescents. J. Mot. Behav. 52 (6), 694–702. 10.1080/00222895.2019.1680949 31650909

[B38] Lubis H. Y. Corwin P. Kharatzadeh M. Kessler P. (2025). Comprehensive injury prevention strategies in gymnastics: a systematic review of biomechanical and training-related risk factors. INSPIREE Indones. Sport Innov. Rev. 6 (02), 106–116. 10.53905/inspiree.v6i02.148

[B39] Ma D. Wang K. Silva R. M. Xu Q. Zhao Z. (2024). A weekly session of jumping interval training effectively enhances aerobic, anaerobic, and jumping performance in aerobic gymnastics. J. Hum. Kinet. 97, 197–211. 10.5114/jhk/193536 PMC12127922 40463311

[B40] Marcolin G. Rizzato A. Zuanon J. Bosco G. Paoli A. (2019). Expertise level influences postural balance control in young gymnasts. J. Sports Med. Phys. Fit. 59 (4), 593–599. 10.23736/s0022-4707.18.08014-3 29845831

[B41] Moeskops S. Oliver J. L. Radnor J. M. Haff G. G. Myer G. D. Ramachandran A. K. (2024). Effects of neuromuscular training on muscle architecture, isometric force production, and stretch-shortening cycle function in trained young female gymnasts. J. Of Strength & Cond. Res. 38 (9), 1640–1650. 10.1519/jsc.0000000000004856 39016320

[B42] Omorczyk J. Wrześniewski K. Staszkiewicz R. Puszczałowska-Lizis E. (2021). Postural stability at different heights as well as in natural standing position and during tandem stance in female athletes who practice artistic gymnastics. Acta Bioeng. Biomechanics 23 (3), 155–162. 10.37190/abb-01869-2021-03 34978314

[B43] Opala-Berdzik A. Głowacka M. Juras G. (2021). Postural sway in young female artistic and acrobatic gymnasts according to training experience and anthropometric characteristics. BMC Sports Sci. Med. Rehabilitation 13 (1), 11. 10.1186/s13102-021-00236-w PMC7881629 33579356

[B44] Peterson M. L. Christou E. Rosengren K. S. (2006). Children achieve adult-like sensory integration during stance at 12-years-old. Gait & Posture 23 (4), 455–463. 10.1016/j.gaitpost.2005.05.003 16002294

[B45] Petran D. Potop V. Mihai I. Eshtaev A. Damian M. Toma G. (2023). Determining the influence of anthropometric indices on balance parameters in young female gymnasts. Pedagogy Phys. Cult. Sports 27 (6), 515–526. 10.15561/26649837.2023.0610

[B46] Potop V. Grad R. Omelyanchik O. Begajlo M. Boloban V. (2014). Element nodes of sports equipment double back flip factions and double back flip hunched performed gymnast in floor exercise. Pedagogy Phys. Cult. Sports 7, 23–31. 10.6084/m9.figshare.1015381

[B47] Qualisys AB (2011). Qualisys Track Manager: User Manual. Gothenburg: Qualisys AB.

[B48] Robertson D. G. E. Dowling J. J. (2003). Design and responses of butterworth and critically damped digital filters. J. Electromyogr. Kinesiol. 13 (6), 569–573. 10.1016/s1050-6411(03)00080-4 14573371

[B49] Rössler R. Wagner J. Knaier R. Rommers N. Kressig R. W. Schmidt-Trucksäss A. (2024). Spatiotemporal gait characteristics across the adult lifespan: reference values from a healthy population – analysis of the COmPLETE cohort study. Gait & Posture 109, 101–108. 10.1016/j.gaitpost.2024.01.005 38290395

[B50] Sands W. A. Mcneal J. R. Jemni M. Penitente G. (2011). Thinking sensibly about injury prevention and safety. Sci. Gymnast. J. 3 (3), 43–58. 10.52165/sgj.3.3.43-58

[B51] Šarabon N. Kozinc Ž. Perman M. (2021). Establishing reference values for isometric knee extension and flexion strength. Front. Physiology 12, 767941. 10.3389/fphys.2021.767941 PMC8554160 34721087

[B52] Semenov D. Shlyakhtov V. Rumyantsev A. (2021). Kinematic analysis as the basis for training strategy in gymnastics. BIO Web Conf. 29, 01012. 10.1051/bioconf/20212901012

[B53] Senior D. (2004). Qualisys Track Manager: User Manual. *Laboratory Memorandum. National Research Council Canada. Institute for Ocean Technology* . 10.4224/8896115

[B54] Silva B. Leite I. Gomes C. Campos M. Sousa F. Ávila-Carvalho L. (2025). The impact of landings and ground reaction forces in artistic gymnastics: a systematic review. Sci. Gymnast. J. 17 (1), 17–34. 10.52165/sgj.17.1.17-34

[B55] Slater A. Campbell A. Smith A. Straker L. (2015). Greater lower limb flexion in gymnastic landings is associated with reduced landing force: a repeated measures study. Sports Biomech. 14 (1), 45–56. 10.1080/14763141.2015.1029514 25895434

[B56] Sleeper M. D. Kenyon L. K. Casey E. (2012). Measuring fitness in female gymnasts: the gymnastics functional measurement tool. Int. Journal Sports Physical Therapy 7 (2), 124–138. 22530187 PMC3325636

[B57] Standing R. J. Maulder P. S. Best R. Berger N. J. A. (2019). The influence of maturation on functional performance and injury markers in Male youth. Cogent Med. 6 (1), 1632017. 10.1080/2331205x.2019.1632017

[B58] Steinberg N. Elbaz L. Dar G. Nemet D. Eliakim A. (2025). Physiological characteristics of young (9-12 years) and adolescent (≥13 years) rhythmic, acrobatic, and artistic female gymnasts. Sports Health A Multidiscip. Approach 17 (6), 1132–1139. 10.1177/19417381251314077 PMC11795574 39905597

[B59] Taneja K. Singla D. Basista R. Choudhury S. (2023). Effect of exercise training on physical fitness among gymnastic athletes: systematic review and metanalysis. Med. Res. Archives 11 (12). 10.18103/mra.v11i12.4780

[B60] Tudor-Locke C. Aguiar E. J. Han H. Ducharme S. W. Schuna Jr J. M. Barreira T. V. (2019). Walking cadence (steps/min) and intensity in 21–40 year olds: CADENCE-adults. Int. J. Behav. Nutr. Phys. Activity 16 (1), 8. 10.1186/s12966-019-0769-6 30654810 PMC6337834

[B61] Vielemeyer J. Müller R. Staufenberg N.-S. Renjewski D. Abel R. (2021). Ground reaction forces intersect above the center of mass in single support, but not in double support of human walking. J. Biomechanics 120, 110387. 10.1016/j.jbiomech.2021.110387 33798969

[B62] Wade M. Campbell A. Smith A. Norcott J. O’ Sullivan P. (2012). Investigation of spinal posture signatures and ground reaction forces during landing in elite female gymnasts. J. Appl. Biomechanics 28 (6), 677–686. 10.1123/jab.28.6.677 22661081

[B63] Webster K. E. Wittwer J. E. Feller J. A. (2005). Validity of the GAITRite® walkway system for the measurement of averaged and individual step parameters of gait. Gait & Posture 22 (4), 317–321. 10.1016/j.gaitpost.2004.10.005 16274913

